# Monitoring Glucocorticoid Receptor in Plasma-derived Extracellular Vesicles as a Marker of Resistance to Androgen Receptor Signaling Inhibition in Prostate Cancer

**DOI:** 10.1158/2767-9764.CRC-23-0362

**Published:** 2023-12-13

**Authors:** Emanuela Gentile, Andrew W. Hahn, Jian H. Song, Anh Hoang, Peter D. A. Shepherd, Sumankalai Ramachandran, Nora M. Navone, Eleni Efstathiou, Mark Titus, Paul G. Corn, Sue-Hwa Lin, Christopher J. Logothetis, Theocharis Panaretakis

**Affiliations:** 1 Department of GU Medical Oncology, MD Anderson Cancer Center, Houston, Texas.; 2 Department of Translational Molecular Pathology, MD Anderson Cancer Center, Houston, Texas.

## Abstract

Disease progression following androgen ablation was shown to be associated with upregulation of the glucocorticoid receptor (GR). Longitudinal monitoring of GR expression in circulating extracellular vesicles (EV) may reflect changes in the tumor cell and facilitates detection of acquired resistance. We utilized LNCaP, LREX cells and a patient-derived xenograft, MDA PDX 322-2-6a, for *in vitro* and *in vivo* experiments. Plasma-derived EVs were isolated from patients with localized high-risk prostate cancer undergoing androgen ablation. The mRNA levels of GR in EVs and their responsive genes were detected by transcriptome analysis, qRT-PCR and the protein levels by Western blot analysis. We detected changes in GR expression at mRNA and protein levels in EVs derived from LNCaP and LREX cells in *in vitro* studies. In *in vivo* experiments, LNCaP and the PDX MDA 322-2-6a–bearing mice were treated with enzalutamide. GR levels in plasma-derived EVs were increased only in those tumors that did not respond to enzalutamide. Treatment of mice bearing enzalutamide-resistant tumors with a GR inhibitor in combination with enzalutamide led to a transient pause in tumor growth in a subset of tumors and decreased GR levels intracellular and in plasma-derived EVs. In a subgroup of patients with high-risk localized prostate cancer treated with androgen signaling inhibition, GR was found upregulated in matching tissue and plasma EVs. These analyses showed that GR levels in plasma-derived EVs may be used for monitoring the transition of GR expression allowing for early detection of resistance to androgen ablation treatment.

**Significance:**

Longitudinal monitoring of GR expression in plasma-derived EVs from patients with prostate cancer treated with androgen signaling inhibitors facilitates early detection of acquisition of resistance to androgen receptor signaling inhibition in individual patients.

## Introduction

Liquid biopsies have been receiving increasing interest as a less invasive approach than solid tumor biopsies to monitor disease progression and tumor response to therapy (1, 2). In recent years, analysis of plasma-derived extracellular vesicles (EV), a component of the liquid biopsies, has demonstrated significant potential as a source of prognostic and predictive biomarkers (3). EVs are nanosized lipid bilayer vesicles that contain proteins, RNAs, and bioactive lipids derived from immune and non-immune components of the tumor microenvironment. Proteins and RNAs enriched in EVs may reflect specific changes in the disease stage and response to therapy (4–6). Cancer cells secrete an elevated number of EVs and mediate homotypic and heterotypic intercellular communication both at local and metastatic sites (7, 8). We have recently shown that RNA profiles of plasma-derived EVs provide molecular information on the bone tumor microenvironment in patients with bone metastatic prostate cancer (9). Longitudinal profiling of EVs may detect dynamic biological changes associated with tumor growth and disease progression (4). As an example of this, Del Re and colleagues demonstrated the detection the AR-V7 splice variants in exosomes derived from patients treated with androgen signaling inhibition (ASI; ref. 10). Because bone is the predominant site of prostate cancer metastases and serial monitoring of bone tumor biopsies poses numerous challenges, the ability to analyze EVs in a liquid biopsy as a “proxy” measurement of the bone tumor microenvironment potentially offers significant clinical utility.

The cornerstone of treatment for men with advanced prostate cancer is androgen deprivation therapy (ADT) with luteinizing hormone-releasing hormone (LHRH) analogs to deplete endocrine levels of testosterone and further intensification with ASI to target adrenal and paracrine androgen signaling. However, resistance to ASI frequently emerges over time (11). One proposed mechanism of acquired resistance to ADT and ASI is induction of glucocorticoid receptor (GR) expression (2). Arora and colleagues demonstrated that GR is upregulated upon ADT and/or ASI, and this induction leads to tumor growth (2). The role of GR in supporting the growth of castration-resistant prostate cancer was further demonstrated by Puhr and colleagues, showing that GR expression is upregulated in metastatic lesions and significantly increased upon long-term abiraterone or enzalutamide (ENZA) treatment (12). At present, GR expression is determined by IHC staining in tissue biopsies. In this study, we sought to develop a method to detect GR in liquid biopsies that could be applied longitudinally during ASI treatment to facilitate early detection of resistance and inform novel therapies strategies.

Herein, we demonstrate that plasma-derived EVs can be used to longitudinally monitor changes in the androgen receptor (AR) and glucocorticoid receptor (GR) mRNA levels in response to ASI in preclinical models and in clinical samples from patient receiving ASI. In mouse models, increased GR levels in the plasma EVs correlated with resistance to ENZA *in vivo*. Subsequent targeting of GR in the ENZA-resistant tumors led to tumor inhibition. Analysis of EVs transcripts from plasma samples from patients treated with ASI revealed the emergence of GR upregulation. Collectively, these data demonstrate that liquid biopsy analysis of plasma-derived EVs can be used to monitor GR-mediated resistance to ASI and suggest that targeting GR represents a valid therapy strategy.

## Materials and Methods

### Cell Line Cultures and Reagents

The LNCaP human prostate cancer cell line was maintained in RPMI medium supplemented with 10% FBS and 1% penicillin/streptomycin. LREX cells were kindly provided by Charles Sawyers Lab and were maintained in RPMI with l-glutamine and HEPES buffer supplemented with 20% FBS (Omega Scientific Inc) and 1% penicillin/streptomycin. LREX cells were cultured continually in 1 µmol/L ENZA. All cell lines were maintained at 37°C and 5% CO_2_ and grown to 70% confluency. Routine testing for *Mycoplasma* contamination was performed. Cell line purity was confirmed by fingerprinting analysis by the MD Anderson Cancer Center Core Facility in Department of Systems Biology. enzalutamide (ENZA) and mifepristone were purchased from MedChem Express LLC.

### EVs Isolation From Cell Lines

To collect EVs from cell supernatants, all cell lines were cultured in EV-depleted medium. To deplete EVs from culture medium, complete medium (+10% or 20% FBS) was centrifuged overnight at 30,000 rpm followed with filtration with sterile bottle-top vacuum filters with a pore size of 0.22 µm. Cells were cultured in 3-layer flasks and cell supernatant was collected every 2–3 days, centrifuged at 1,400 rpm for 10 minutes, filtered and stored at −20°C. EVs were isolated from collected supernatant in two consecutive ultracentrifuges steps. The first step was at 30,000 rpm 4°C for 2 hours and second at 35,000 rpm 4°C for 2 hours. Pellets were resuspended in different buffers depending on the assay: for Nanosight [nanoparticle tracking analysis (NTA)]: approximately 20 mL of PBS with 2 mmol/L Ethylenediaminetetraacetic acid (EDTA); for Cell treatments: approximately 20 mL of sterile medium with 2 mmol/L EDTA; for protein: approximately 20 mL of 1X RIPA; for RNA extraction: 700 mL of QIAzol Lysis Reagent. Resuspended or lysed EVs were stored in −80°C until future experiments.

### NTA

For the determination of particle concentration and size, NS500 (NanoSight Limited), equipped with an 8 mega pixel camera (Andor Technology) and a 405 nm laser, was used to measure the size and determine concentration of EVs. NTA v3.2 software (NanoSight Limited) was used for both data acquisition and analysis. The duration of each video was 5 × 1 minute. During the analysis procedure, the camera level was 14 and the detection threshold was 3. Samples were diluted from 1: 100 to 1:1,000 in PBS 2 mmol/L EDTA.

### Gene Expression and Protein Expression in Cells and Derived EVs

RNA was isolated from cells and EVs using RNAeasy micro kit (Qiagen) according to manufacturer's protocols and RNA concentration was determined using a NanoPhotometer. Two-step RT-PCR was performed for gene expression analysis. In the first step, 100 ng of total RNA (concentration 10 ng/µL) from cells or EVs was used to synthesize cDNA with SuperScript IV VILO Master Mix (Thermo Fisher Scientific) according to manufacturer's protocols in a thermocycler; in the second step, 10 µL of cDNA were mixed with PowerUp SYBR Green Master Mix and specific target primers at concentration of 450 nmol/L (KiCqStart Syber green primers, Sigma) for gene expression analysis in RT-PCR instruments (Applied Biosystems QuantStudio Real-Time PCR). Standard cycling mode was running for 40 cycles according to PowerUp SYBR Green Master Mix manufacturer's protocols. Syber green primer sequences used in this project are: **Human NR3C1**, reverse primer sequence-GATTTTCAACCACTTCATGC-forward primer sequence -ACTGCTTCTCTCTTCAGTTC-; **Human KLK3**, reverse primer sequence -AGAATCCTCTGGTTCAATG- forward primer sequence – TATGAGCCTCCTGAAGAATC-. **Human β-actin**, reverse primer sequence -GCCCACATAGGAATCCTTCTGAC- -AGGCACCAGGGCGTGAT- was used as a control for the cells and **Human 18s**, -reverse primer sequence TTATCTAGAGTCACCAAGCC-, forward primer sequence -CAGTTATGGTTCCTTTGGT- as control for RNA from EVs. Cells and EVs pellets were lysed using 1 x RIPA buffer (containing 50 mmol/L Tris, 150 mmol/L NaCl, 1 mmol/L EDTA, 1% IGEPAL, 1% glycerol), supplemented with Complete Protease Inhibitor Cocktail (Roche), PhosSTOP Phosphatase Inhibitor Cocktail (Roche), 100 mmol/L vanadate (Invitrogen Life Technologies), and 1 mmol/L dithiothreitol (Sigma-Aldrich). Protein concentration was determined with Bradford assay using Bradford solution (Bio-Rad). Proteins were separated in 10% or 12% Bis-Tris gels and transferred to polyvinylidene difluoride membranes using the Trans-Blot Turbo System. Membrane was incubated with primary antibodies in the appropriate dilutions (1:500–1:1,000) overnight at 4°C. The following antibodies were used: GR from Cell Signaling (D6H2L), AR from Santa Cruz Biotechnology (441), KLK3 from cells signaling (D11E1), and GAPDH from Cell Signaling (14C10). Horseradish peroxidase–conjugated antibodies in 1:1,000 dilution was used as secondary antibodies for 1 hour at room temperature. Image manager system was used for proteins detection and analysis (KwikQuant, Kindle Biosciences, LLC)

### Transfection with Short Hairpin RNA Lentiviral Particles

Mission custom short hairpin RNA (shRNA) lentiviral particles were purchased from Sigma (Sigma Millipore). The sequence of the NR3C1 shRNA-II was as follows: 5′-GTGTCACTGTTGGAGGTTATT-3′; a MISSION pLKO.1-puro non-Target shRNA Control Transduction Particles was used as a negative control. LNCaP cells were seeded at 4 × 10^5^ cells/well in a 6-well plate prior to lentiviral particles infection and incubated with 2 mL of complete medium without antibiotics for 24 hours. The day after the cells were previously coated with 8 µg/mL polybrene (Sigma Millipore) and then infected by the addition of the shRNA lentiviral particles at a multiplicity of infection of 10–20; the plates were then gently swirled for mixing and incubated for 24 hours. The virus-containing medium of infected wells was removed, and fresh complete medium was added. On day 3, puromycin selection was added at a final concentration of 5 µg/mL in all wells except the puromycin (−) control, for 3–5 days. qRT-PCR and Western blot analysis were performed to determine the shRNA interference efficiency (GR primers and GR antibody for Wester blotted reported previously).

### Animal Studies

NOD/SCID mice were provided by MD Anderson Cancer Center, Experimental Radiation Oncology, Department of Veterinary Medicine & Surgery, authorized by the Jackson Laboratory. All animal studies were conducted in accordance with the current regulations and standards of the U.S. Department of Agriculture, the U.S. Department of Health and Human Services and the NIH and were approved by The University of Texas MD Anderson Cancer Center Institutional Animal Care and Use Committee. NOD/SCID mice were subcutaneously injected with LNCaP cells around 5 × 10^6^ cells/mouse mixed with Matrigel 1:1. After the tumor reached the size of 100 mm^3^, mice were treated with 10 mg/kg ENZA (MedChem Express LLC) diluted in 5% DMSO: 40% PEG400: 5% TWEEN-80: 50% WATER. ENZA was administrated by oral gavage 5 days/week. Mifepristone (MedChem Express LLC) was given in combination with ENZA for selected mice at the concentration of 10 mg/kg and administrated by oral gavage 5 days/week. Tumor growth was recorded twice weekly while on treatment for approximately 50 days. At the end of experiment, mice were euthanized by asphyxiation with carbon dioxide and tumor and blood were collected for future analysis.

### PDX Model Studies

MDA patient-derived xenografts (PDX) were developed in the laboratory of Dr. Navone at the “Prostate Cancer Patient Derived Xenografts Program”, Department of Genitourinary Medical Oncology, MD Anderson Cancer Center, and the David H. Koch Center for Applied Research of Genitourinary Cancers. PDXs were established following previous described procedures and propagated as subcutaneous xenografts in 6 to 8 weeks old male NOD/SCID gamma male mice (Experimental Radiation Oncology, MD Anderson Cancer Center). Maintenance of PDXs in mice was approved by the Institutional Animal Care and Use Committee of The University of Texas MD Anderson Cancer Center. MDA PDX 322-2-6a (Negative GR) were implanted and treated with ENZA (MedChem Express LLC) 10 mg/kg when the tumor size reached 200 mm^3^. ENZA was diluted in 5% DMSO: 40% PEG400: 5% TWEEN-80: 50% WATER and administrated by oral gavage 5 days/week. Mifepristone (MedChem Express LLC) was given in combination with ENZA for selected mice at the concentration of 10 mg/kg and administrated by oral gavage 5 days/week. Tumor growth was recorded twice weekly while on treatment for around 50 days. At the end of experiment, mice were euthanized by asphyxiation with carbon dioxide and tumor and blood were collected for future analysis.

### IHC Procedure for GR Antibody on Mouse and Human Tissues

We performed IHC analyses of GR expression in mouse tissue specimens obtained from MDA PDX 322-2-6a at the end of the experiment and from prostate tissue specimens obtained by prostatectomy. GR (Clone 41; Biosciences – catalog no.: 611226) was used at dilution 1/150 (mouse tissues) and 1/300 (prostate human tissues) for 1 hour at room temperature, washed and incubated with Envision Dual Link System (DAKO, catalog no.: K4061) for 30 minutes at room temperature. After that the slides were covered in 3,3ʹ-Diaminobenzidine solution (DAKO, catalog no.: K3468) and incubate for 10 minutes, and counterstained with CAT Hematoxylin (Biocare Medical, catalog no.: CATHE-G) and Bluing Reagent (Thermo Fisher Scientific, catalog no.: 22-050-114). Dehydration through 95% ethanol, 100% ethanol, was followed by clarification in xylene. At the end, coverslip were added with mounting medium. For semiquantitative/quantitative analysis, the stained slides were digitalized in an Aperio AT2 scanner (Leica Biosystems) and quantified using ImageJ software.

### Total EVs Isolation From Human Plasma for Protein Isolation and NanoSight Analysis

A total of 500 mL of plasma samples was thawed in ice and mixed with 500 µL of cold PBS before centrifuge at 12,000 × *g* for 45 minutes at 4°C to remove cellular debris. The supernatant was transferred to an ultracentrifuge tube, and 9 mL of cold PBS was added, and samples were ultra-centrifuged for 2 hours at 35,000 rpm. After centrifuge, the EV pellet was directly resuspended in 20 µL of 1x RIPA for protein lysis. Protein concentration was determined with Bradford assay using Bradford solution (Bio-Rad). A total of 20 µL of PBS 2 mmol/L EDTA for NTA. Samples were stored at −80°C.

### Total EVs Isolation and RNA Extraction From Human Plasma

To isolate EVs from patient's samples, 500 µL of human plasma were thawed on ice and centrifuge at 500 × *g* for 10 minutes at 4°C. For 500 mL of human plasma, 100 mL of total exosome isolation kit (Invitrogen, by Thermo Fisher Scientific) was added and homogenized by vortexing. After 30 minutes of incubation on ice, the samples were centrifuged for 10 minutes at 10,000 × *g* at room temperature and the EV pellet was resuspended in 100 µL of PBS. Total RNA extraction from isolated plasma-derived EVs started by adding 700 µL of Qiazol Lysis Reagent and homogenizing by vortex. miRNeasy Micro Kit (Qiagen, catalog no.: 217084) was used, according to the manufacturer's instructions. The RNA quantification was performed with a NanoPhotometer. After isolation, RNA purity and quantification analysis were performed by an RNA 6000 Pico kit on the Agilent Bioanalyzer 2100 system.

### Transcriptome Analysis: Affymetrix Clariom D Assay

RNA profiling of EVs was performed by Sequencing and Microarray Facility at the University of Texas MD Anderson Cancer Center. Affymetrix's Clariom D assay provided a detailed view of the transcriptome with all known coding and noncoding splice variants. Transcriptome Analysis Console (TAC) Software was used to analyze and visualize global expression patterns of genes, exons, pathways, and alternative splicing events.

### Steroid Derivatization Using Hydroxylamine Hydrochloride and LC/MS-MS Quantification

A total of 10 µL of C-13 testosterone was added as the internal standard to 500 µL of previously frozen plasma samples and kept on ice. Liquid-liquid extraction was performed using Methyl t-butyl ether (MTBE; 3.0 mL). After centrifugation, the organic layer was then collected. The sample was then derivatized using methanol (100 µL) and hydroxylamine hydrochloride (1.5 mol/L; 100 µL) heated at 65°C for 60 minutes. An 8-point standard calibration curve for the androgens was created in charcoal-stripped plasma. The samples were then transferred into LC/MS vials and analyzed (Agilent 6495 Mass Spectrometer with Jet Stream Technology and ESI source). Standard compounds and Internal Standard compounds were obtained from Sigma: testosterone (Steraloids, A6950-000), cortisol (Steraloids, Q3880-000); testosterone (^3^C_13_) (Isosciences, S6066); cortisol (^2^H_4_) (Isosciences, S7193).

### Statistical Analysis

For the *in vitro* and *in vivo* studies and for the patient's samples, GraphPad Prism 9 was used for statistical analysis. Data are presented as means ± SEM. Unpaired two-tailed Student *t* tests were used to compare the means of two samples. Differences were considered statistically significant when *P* < 0.05. Gene set enrichment analysis (GSEA) was performed using the GSEA v4.3.2 software. Hallmarks and Kyoto Encyclopedia of Genes and Genomes analysis were used for enrichment of Androgen Response and Prostate cancer signaling pathway-related genes. All the gene set files were obtained from GSEA website (www.broadinstitute.org/gsea/). Enrichment map was used for visualization of the GSEA results. Enrichment score and FDR value were applied to sort Androgen Response and Prostate Cancer pathway enriched. The IPA system (version 90348151, Ingenuity Systems; Qiagen China Co., Ltd.) was used for bioinformatics analysis, which included canonical pathway analysis. The z score is generated on the basis of hypergeometric distribution and the logarithm of the significance level is obtained by right-tailed Fisher exact test.

### Data Availability

All data are available in the main text or the Supplementary Materials and Methods. Further information related to the materials is available from the corresponding author.

## Results

### GR is Detected in EVs of ENZA-treated Cells *In Vitro*

**FIGURE 1:**
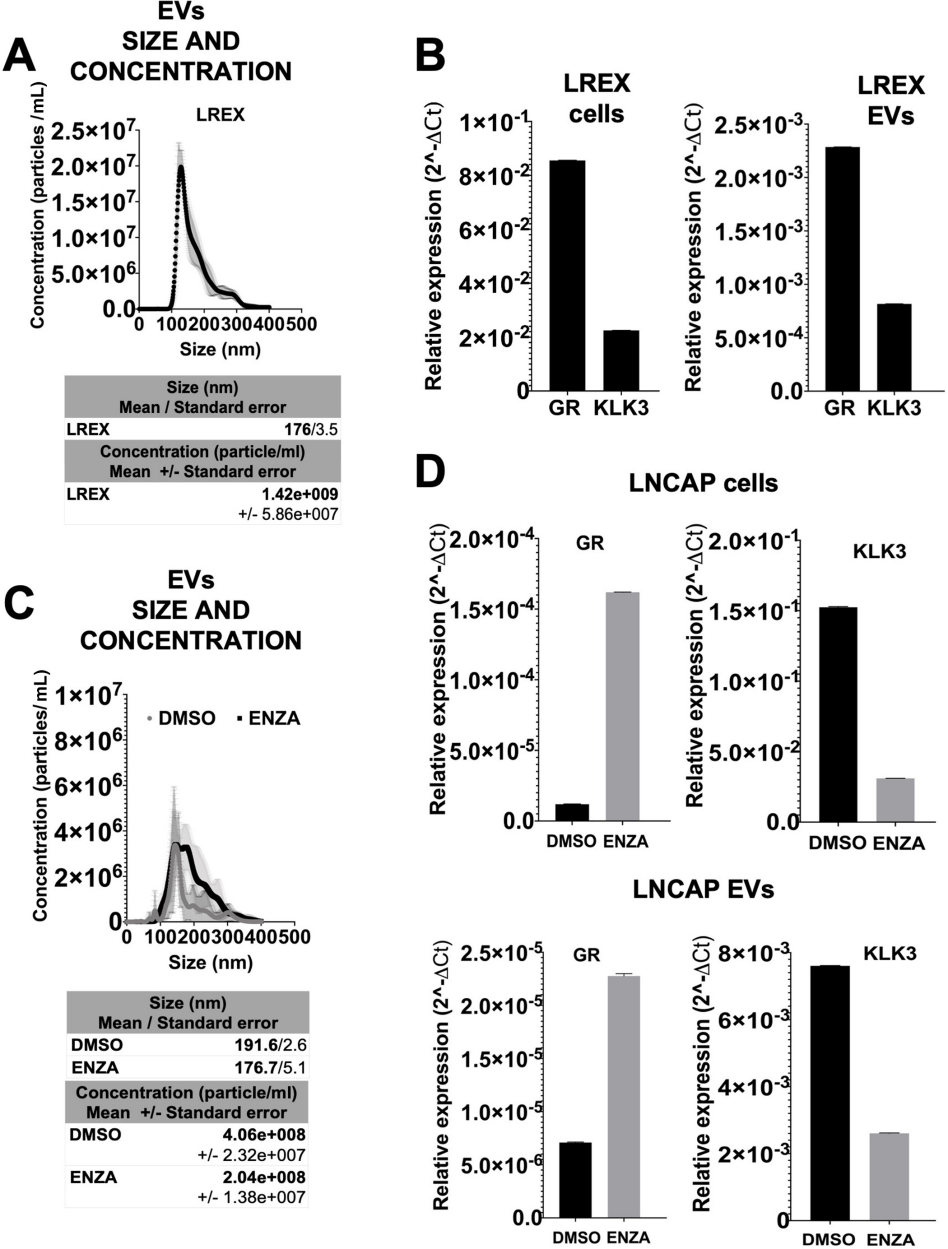
ENZA-induced GR RNA changes in EVs *in vitro.***A,** Characterization of LREX EVs using NTA. **B,** qRT-PCR for GR and KLK3 in RNAs isolated from LREX cells and LREX EVs, respectively. **C,** LNCaP EVs characterization using NTA. **D,** qRT-PCR for the expression of GR and KLK3 in RNAs isolated from cells and EVs after LNCaP were treated for 5 days with 1 µmol/L ENZA. Actin and 18s are used for total cellular RNA and total RNA from EVs normalization, respectively.

It was previously shown that GR expression was upregulated in LREX, an established ENZA-resistant cell line that expresses high levels of AR but not KLK3 (PSA) due to ASI (2). We used LREX cells to examine whether these transcripts are enriched in the EVs isolated from the conditioned medium of these cells. EVs isolated from these LREX cells, characterized by size and concentration using NTA (Fig. 1A), showed KLK3 and GR levels proportional to those expressed in the cells by qRT-PCR (Fig. 1B) and Western blot analysis (Supplementary Fig. S1A). We then treated LNCaP cells with 1 µmol/L ENZA for 5 days, to induce GR expression, as shown previously (2, 12). NTA of EVs isolated from these LNCaP cells, revealed that the size of EVs secreted by LNCaP cells were similar with or without ENZA treatment, but the EVs concentration was lower compared with control DMSO-treated cells (DMSO; Fig. 1C). RNA and proteins were extracted from cells and cell-derived EVs for qRT-PCR and Western blot analysis, respectively. In ENZA-treated LNCaP cells, GR mRNA was induced and KLK3 mRNA was decreased, as measured by qRT-PCR, compared with control LNCaP cells in both cellular RNA and RNA extracted from EVs (Fig. 1D). Similar results were obtained in protein levels as shown by Western blot analysis (Supplementary Fig. S1B). Western blot analysis of cell lysates and EVs showed different enrichment of TSG101, an EV marker that is used as loading control (Supplementary Fig. S1A and S1B). High Sensitivity RNA ScreenTape EUKARYOTIC RNA ANALYSIS was also used to demonstrate that the quality, quantity, and sizes of total RNA and RNA extracted from EVs, from LNCaP cells treated with or without ENZA, were similar (Supplementary Fig. S2A). These results suggest that GR transcript can be detected in EVs, and its level correlates with changes in the cellular GR RNA level.

**FIGURE 2:**
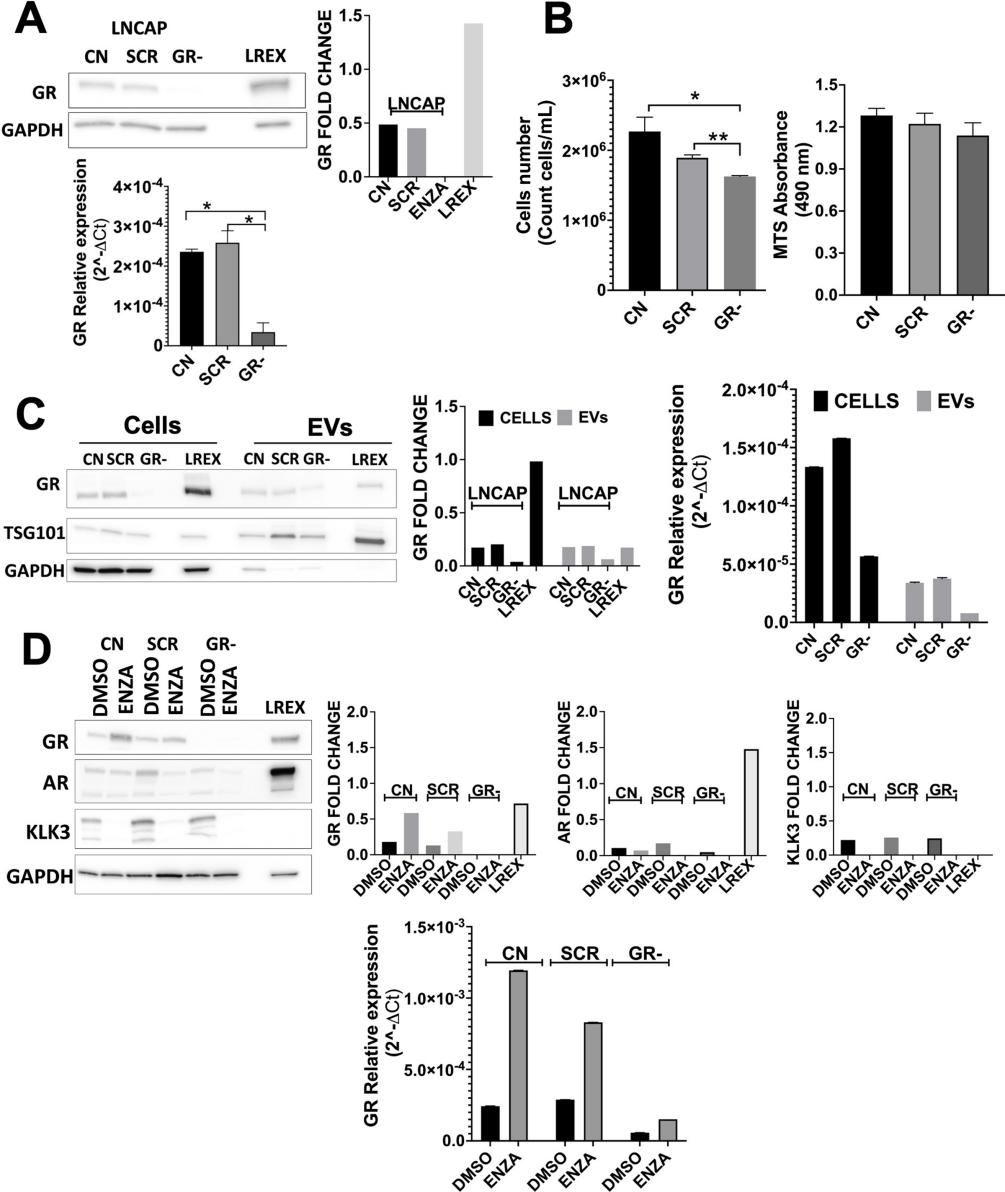
GR knockdown *in vitro.***A,** Immunoblot analysis of the indicated proteins in LNCaP (LREX were used as GR expression control) cells after transduction with shGR-2 (GR−). qRT-PCR for GR (GR− vs. CN, *, *P* = 0.0143; GR− vs. SCR, *, *P* = 0.0276). **B,** LNCaP cells count (GR− vs. CN, *, *P* = 0.0341; GR− vs. SCR, **, *P* = 0.0036) and MTS after knockdown. **C,** Comparison of GR expression in cells and EVs derived by immunoblot analysis and qRT-PCR of GR expression. **D,** Immunoblot analysis and qRT-PCR of GR expression on LNCaP cell GR knockdown (GR−) after treatment for 5 days with 1 µmol/L ENZA.

To further determine whether the levels of GR transcript in EVs, reflect the changes in the cellular GR, we transduced LNCaP cells with two puromycin-inducible lentiviral shGR vectors (shGR-I, shGR-II; Supplementary Fig. S3A and S3B). Western blotting and qRT-PCR confirmed the knockdown efficiency of LNCaP cells transfect with shGR-II (GR−) compared with control LNCaP cells (CN) and LNCaP transduced with the control lentiviral vector (SCR; Fig. 2A). GR knockdown resulted in decreased cell number and cell proliferation of LNCaP cells (Fig. 2B), consistent with a previous report that GR plays a role in cancer cell proliferation (12). Knockdown of GR decreased the expression of GR in LNCaP cells as well as in the EVs by Western blot analysis and qRT-PCR compared with the EVs from control LNCaP cells (CN) and LNCaP transduced with scramble shRNA (SCR; Fig. 2C). Next, we examined whether the response of GR knockdown impacted the response of cells to ENZA treatment. As demonstrated previously, ENZA treatment led to an increased expression of GR in LNCaP CN and SCR cells but not in the GR− cells by Western blotting and qRT-PCR (Fig. 2D; Supplementary Fig. S3C). ENZA treatment reduced proliferation and increase cell death in CN, SCR and GR− cells. (Supplementary Fig. S3C and S3D).

### Measuring Changes in GR mRNA Levels in Plasma EVs in LNCaP Tumors *In Vivo*

**FIGURE 3:**
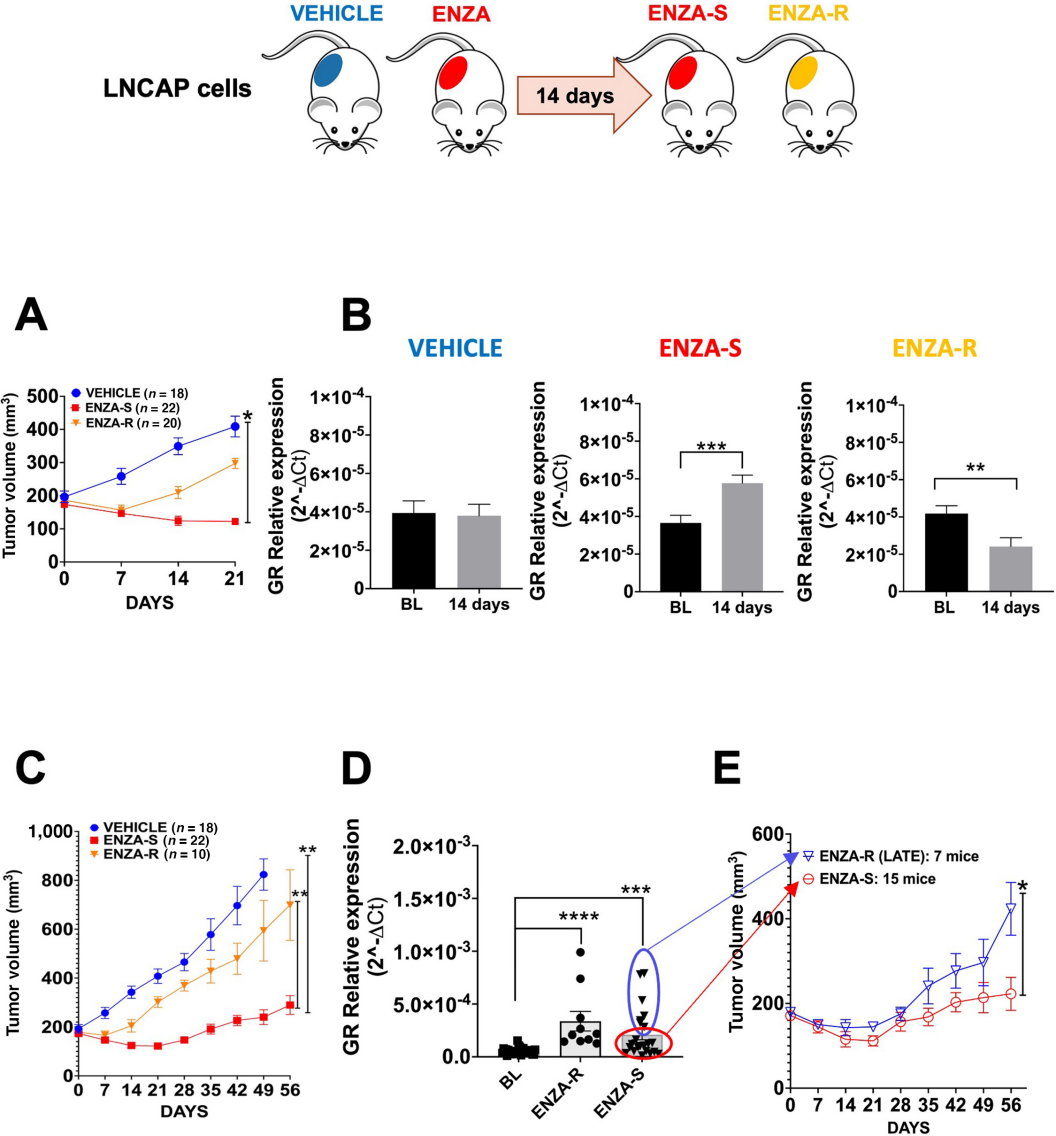
Effect of ENZA on GR in EVs in LNCaP tumors *in vivo*. Schema and treatment planning for *in vivo* experiment with subcutaneously injected LNCAP cells in NOD SCID mice. **A,** Tumor volume VEHICLE, ENZA-S and ENZA-R groups after 14–21 days of treatment (ENZA-S vs. VEHICLE, *, *P* = 0.0160). **B,** qRT-PCR for GR 14 days vs. BL: VEHICLE group (*n* = 18), ENZA-S group (*n* = 22; **, *P* = 0.0083) and ENZA-R group (*n* = 20; ***, *P* = 0.0009). **C,** Final tumor growth analysis of VEHICLE group (*n* = 18) and ENZA-S group (*n* = 22; ENZA-S vs. vehicle, **, *P* = 0.0017) and ENZA-R group (*n* = 10) vs. ENZA-S (**, *P* = 0.0088). **D,** Comparison of qRT-PCR results of GR between BL and ENZA-R and ENZA-S at end of the experiment (ENZA-R, END vs. BL, ****, *P* < 0.0001; ENZA-S, END vs. BL, ***, *P* = 0.0004). **E,** Comparison of tumor volume between ENZA-S and ENZA late resistance mice (ENZA-R LATE) in ENZA-S mice. (*, *P* = 0.0212).

To examine whether changes in GR transcript in EVs could be detected in the plasma of mice bearing LNCaP tumors, LNCaP cells were inoculated subcutaneously in NOD/SCID mice (5 × 10^6^ cell/mouse). At 3 weeks postinjection (tumor size 60–100 mm^3^), the mice were randomized and divided into two groups, (i) VEHICLE (18 mice) or (ii) treated with ENZA (ENZA) 10 mg/kg (50 mice). The mice were treated 5 days per week by oral gavage and tumor growth was monitored weekly. Blood was drawn from these mice at baseline (BL; 200 µL which was sufficient to run qRT-PCRs), at 14 days, and at the end of treatment (END; Fig. 3). EVs were isolated from the plasma and the extracted RNA was subjected to qRT-PCR for the detection of changes in GR expression. qRT-PCR was selected as the method of choice for the detection of GR changes in the remainder of the study due to high sensitivity of the assay compared with relatively larger number of EVs required for Western blotting.

After 14 days, tumor growth showed differential kinetics in response to ENZA. We noticed that in mice treated with ENZA, there one group of animals (= 20) with tumors that decreased in size with treatment (ENZA sensitive, ENZA-S) and a second group ( = 20) with tumors increasing in size (ENZA early resistance, ENZA-R; Fig. 3A). qRT-PCR analysis showed that in mice with decreased tumor growth (ENZA-S) in response to ENZA, GR detected in the plasma-derived EVs, was significantly reduced at 14 days compared with BL (Fig. 3B). In contrast, in ENZA-R mice, GR levels were induced in EVs (Fig. 3B). The differences in GR expression in EVs were due to ENZA because they were not observed in the vehicle group at 14 days (Fig. 3B). Mice continued treatment for a total of 56 days and tumor growth size was recorded weekly (Fig. 3C). The group of mice that demonstrated decreased tumor growth (ENZA-S) had low GR expression in EVs compared with the vehicle control (Fig. 3D). The mice that demonstrated increase tumor growth (ENZA-R) had increased GR expression in EVs compared with vehicle control (Fig. 3D). We noticed that 7 of 22 mice in the ENZA-S group showed an increase in tumor volume at the end of the experiment (ENZA-R LATE), consistent with the late acquisition of resistance to ENZA. Notably, the GR levels in EVs from these 7 ENZA-R LATE mice were increased, consistent with GR-depending mechanism (Fig. 3E).

### Measuring Changes in GR mRNA Levels in EVs, in MDA PCa 322-2-6a (PDX model)

**FIGURE 4:**
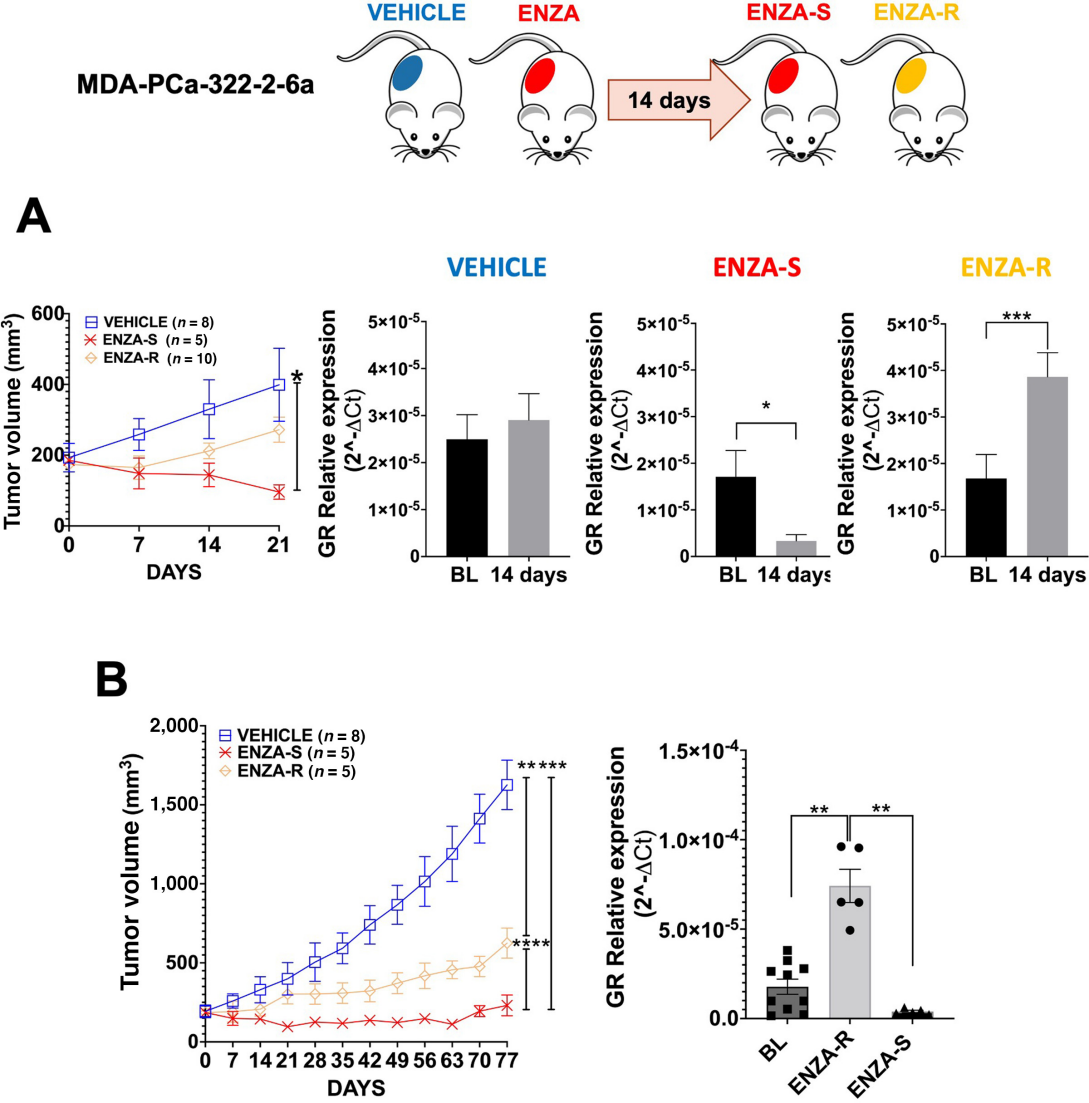
Effect of ENZA on GR in EVs in MDA PCa 322-2-6a tumors *in vivo* (PDX model)*.* Schema and treatment planning for *in vivo* experiment with MDA PCa 322-2-6a tumors. **A,** Tumor volume after 14–21 days of treatment in vehicle group and ENZA-S and ENZA-R (ENZA-S vs. vehicle, *, *P* = 0.0019); qRT-PCR for GR in VEHICLE (*n* = 8; 14 days vs. BL), ENZA-S group (*n* = 5; 14 days vs. BL, *, *P* = 0.0479) and ENZA-R group (*n* = 5; 14 days vs. BL, ***, *P* = 0.0003). **B,** Final tumor growth analysis of VEHICLE group (*n* = 8) and ENZA-R (*n* = 5) and ENZA-S group (= 5; ENZA-R vs. vehicle, **, *P* = 0.0075), (ENZA-S vs. vehicle, ***, *P* = 0.0002), (ENZA-R vs. ENZA-S, ****, *P* ≤0.0001). Comparison of qRT-PCR results of GR between ENZA-R and ENZA-S and BL (ENZA-R vs. BL, **, *P* = 0.0063; ENZA-S vs. ENZA-R,**, *P* = 0.0015).

Compared with cell lines, human PDXs more closely recapitulate the characteristics of the parental tumor. To further establish the feasibility and relevance of using plasma-derived EVs to monitor expression of GR and ENZA resistance, we studied a PDX MDA prostate cancer (PCa) 322-2-6a that is AR+/GR−. MDA PCa 322-2-6a tumors were subcutaneously transplanted in NOD/SCID mice and mice were randomized and divided in two groups when the tumor size reached the range of 100–300 mm^3^ (Fig. 4). Eight mice were treated with vehicle and 20 mice were treated with 10 mg/kg ENZA. The mice were treated 5 days per week by oral gavage and tumor growth was monitored weekly. Blood (200 µL) was drawn from these mice at BL, at 14 days and the end of treatment for exosome isolation.

Similar what was observed with LNCaP tumors, MDA PCa 322-2-6a tumors showed a differential response to ENZA treatment, with a group that responded (ENZA-S = 5 mice) and a group that did not (ENZA-R = 10 mice; Fig. 4A). qRT-PCR analysis showed that the GR mRNA levels in EVs, correlated with the tumor growth after 14 days of ENZA treatment (Fig. 4A). In the ENZA-S group, GR levels in EVs were low compared with BL, while in the ENZA-R group GR levels in EVs was significantly elevated (Fig. 4A). The ENZA-S and ENZA-R mice were treated continually with ENZA with daily oral gavage for 80 days and tumor growth was recorded weekly (Fig. 4B). Overall, ENZA treatment led to significant decrease in tumor volume compared with vehicle group. GR expression in EVs was low in ENZA-S mice and high in ENZA-R mice (Fig. 4B). These data suggest that tumor volume correlates with the plasma derived EVs GR transcript levels quantified by qRT-PCR.

### Targeting of the ENZA-resistant GR+ tumors with a GR Inhibitor Leads to a Decrease in GR Levels in Tumor and Plasma-derived EVs

**FIGURE 5:**
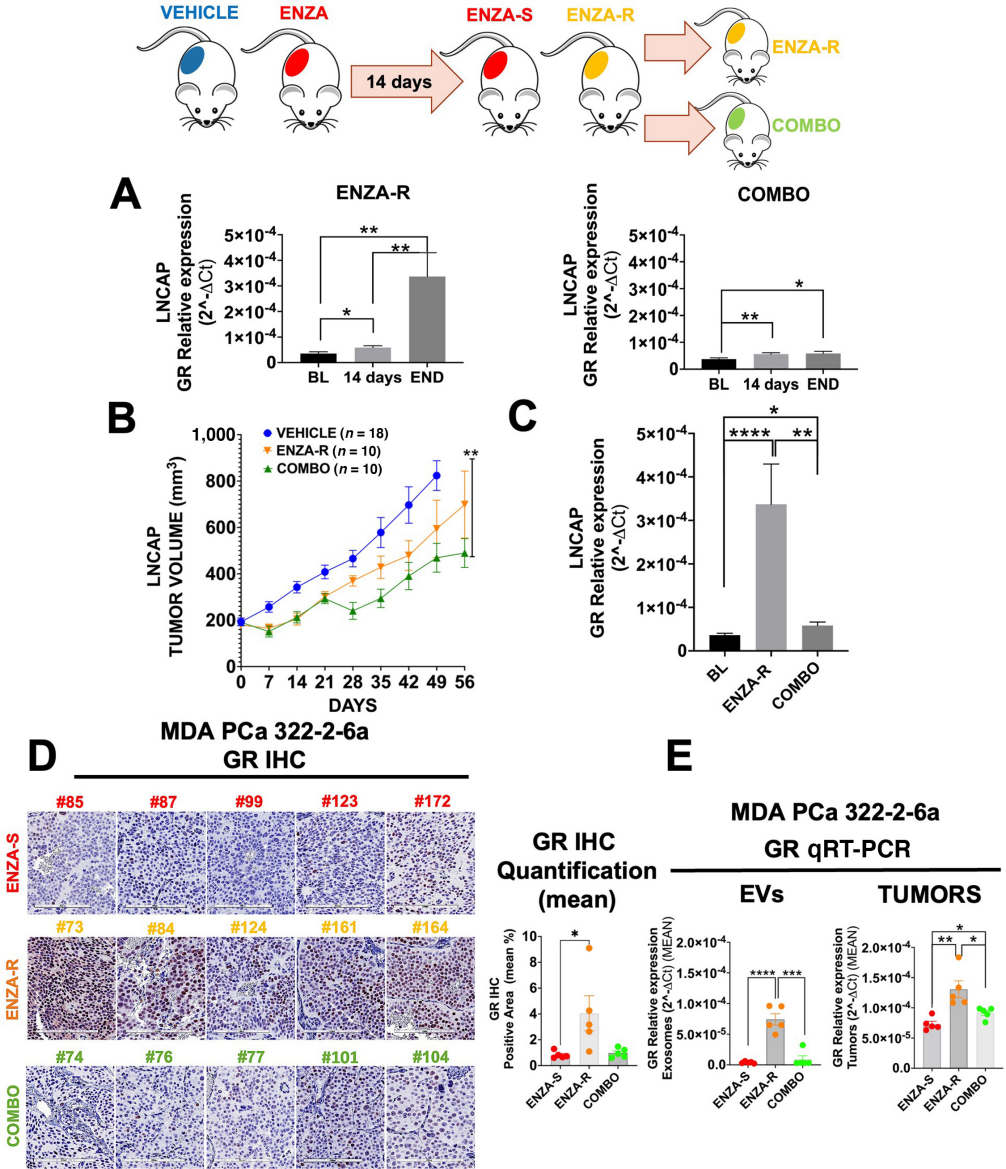
Effect of GR inhibitor in combination with ENZA on ENZA-resistant tumors. Schema and treatment planning for *in vivo* experiment with LNCaP cells subcutaneous injected in NOD SCID mice; ENZA-R group was treated with COMBO (enzalutamide± mifepristone) between 21 and 28 days. **A,** Longitudinal analysis of ENZA-R (14 days vs. BL, *, *P* = 0.0246; end vs. BL, **, *P* = 0.0045; 14 days vs. end, **, *P* = 0.0078) and COMBO (Enzalutamide± Mifepristone; 14 days vs. BL, **, *P* = 0.0096; end vs. BL, *, *P* = 0.0385) from BL to 14 days until the end of the experiment (56 days) in LNCaP *in vivo* experiment. **B,** Final tumor growth analysis in LNCaP *in vivo* experiment (COMBO vs. VEHICLE, **, *P* = 0.0029). **C,** Comparison of GR expression for ENZA-R and COMBO mice in LNCaP *in vivo* experiment (ENZA-R vs. BL, ****, *P* ≤0.0001; COMBO vs. ENZA-R, **, *P* = 0.0077; COMBO, *, *P* = 0.0103). **D,** IHC images and quantification of GR expression between ENZA-S, ENZA-R, and COMBO groups, in MDA PCa 322-2-6a *in vivo* experiment. (ENZA-R vs. ENZA-S, *, *P* = 0.0456; quantification with ImageJ software). **E,** qRT-PCR results of GR for ENZA-S and ENZA-R and COMBO in plasma-derived EVs (ENZA-R vs. ENZA-S, ****, *P* ≤0.0001; COMBO vs. ENZA-R, ***, *P* = 0.0003) and tumors tissues in MDA PCa 322-2-6a *in vivo* experiment (ENZA-R vs. ENZA-S, **, *P* = 0.0045; COMBO vs. ENZA-R, *, *P* = 0.0277; COMBO vs. ENZA-S, *, *P* = 0.0196).

To investigate whether ENZA-induced GR upregulation played a role in ENZA-R tumor growth, we treated the ENZA-R tumors with mifepristone, a GR inhibitor (13–15). Mice inoculated with LNCaP or PDX MDA PCa 322-2-6a, were first treated with ENZA and after 14 days, the ENZA-R (20/42) mice were divided in two groups. One group was maintained with ENZA only treatment and the other group was treated with ENZA plus mifepristone (COMBO; Fig. 5). Treatment with COMBO started between 21 and 28 days after treatment with ENZA and at a tumor size of approximately 300 mm^3^. In LNCaP model, the mice that were continually treated with ENZA alone had increasing levels of GR in the plasma-derived EVs (Fig. 5A) whereas the mice that were treated with the COMBO had low levels of GR throughout the treatment. We found that in COMBO group, tumor growth was attenuated for about 2 weeks before it started growth resumed (Fig. 5B). At the end of the LNCaP experiment, that is, 56 days, GR levels in EVs in COMBO group were low compared with vehicle control group, despite tumor regrowth from COMBO treatment (Fig. 5C), suggesting that resistance to mifepristone is GR-independent. The ENZA plus mifepristone COMBO study was also examined using the MDA PCa 322-2-6a PDX model (Supplementary Fig. S4). As was observed in LNCaP tumors, induction of GR mRNA was detected in plasma-derived EVs, at 14 days posttreatment in the ENZA-R group (10 mice). The ENZA-R mice were divided in two groups, with 5 mice continued treatment with ENZA alone and 5 started on COMBO (Supplementary Fig. S4). At the end of the experiment, the COMBO group showed a longitudinal decreased in GR expression (Supplementary Fig. S4A) and an initial lower tumor growth than the ENZA alone treated group. However, the tumor growth resumed after a transient growth inhibition (Supplementary Fig. S4B). Thus, in both LNCaP and MDA PCa 322-2-6a models, inhibition of GR by mifepristone inhibited tumor growth in ENZA-resistant tumors, but the effect was transient, suggesting AR- and GR-independent resistance mechanics (Supplementary Fig. S4C).

At the end of experiment, tumors from each mouse were collected and IHC staining were performed for GR expression and quantified with Image J software (Fig. 5D). The images and relative quantification indicated that the levels of GR in EVs were low in the ENZA-S group, increased in the ENZA-R and decreased in the COMBO group at the end of experiment (END; Fig. 5D). Consistently, the intracellular levels of GR detected by IHC showed that GR protein levels were significantly reduced by the GR inhibitor mifepristone in the COMBO group indicating that the inhibitor was hitting its target (Fig. 5D). Levels of intracellular GR mRNA (TUMORS) and GR mRNA of EVs correlated with GR protein levels by IHC in the three groups of mice. The ENZA-S tumors had low expression of GR mRNA in the tissues (Fig. 5E) and in the plasma-derived EVs (Fig. 5E), the ENZA-R tumors had proportionally similar higher levels of GR in the tissues and in the plasma-derived EVs, while in the COMBO group GR was inhibited by mifepristone (Fig. 5E).

These data provide further evidence that GR mRNA levels of EVs correlate well with intratumor GR mRNA and protein levels, and this technology can be used as a pharmacodynamic measure for efficacy of targeting GR.

### Measurement of GR mRNA in EVs Isolated from Liquid Biopsy of Patient Samples Reveals Differential Subset of Patients

**FIGURE 6:**
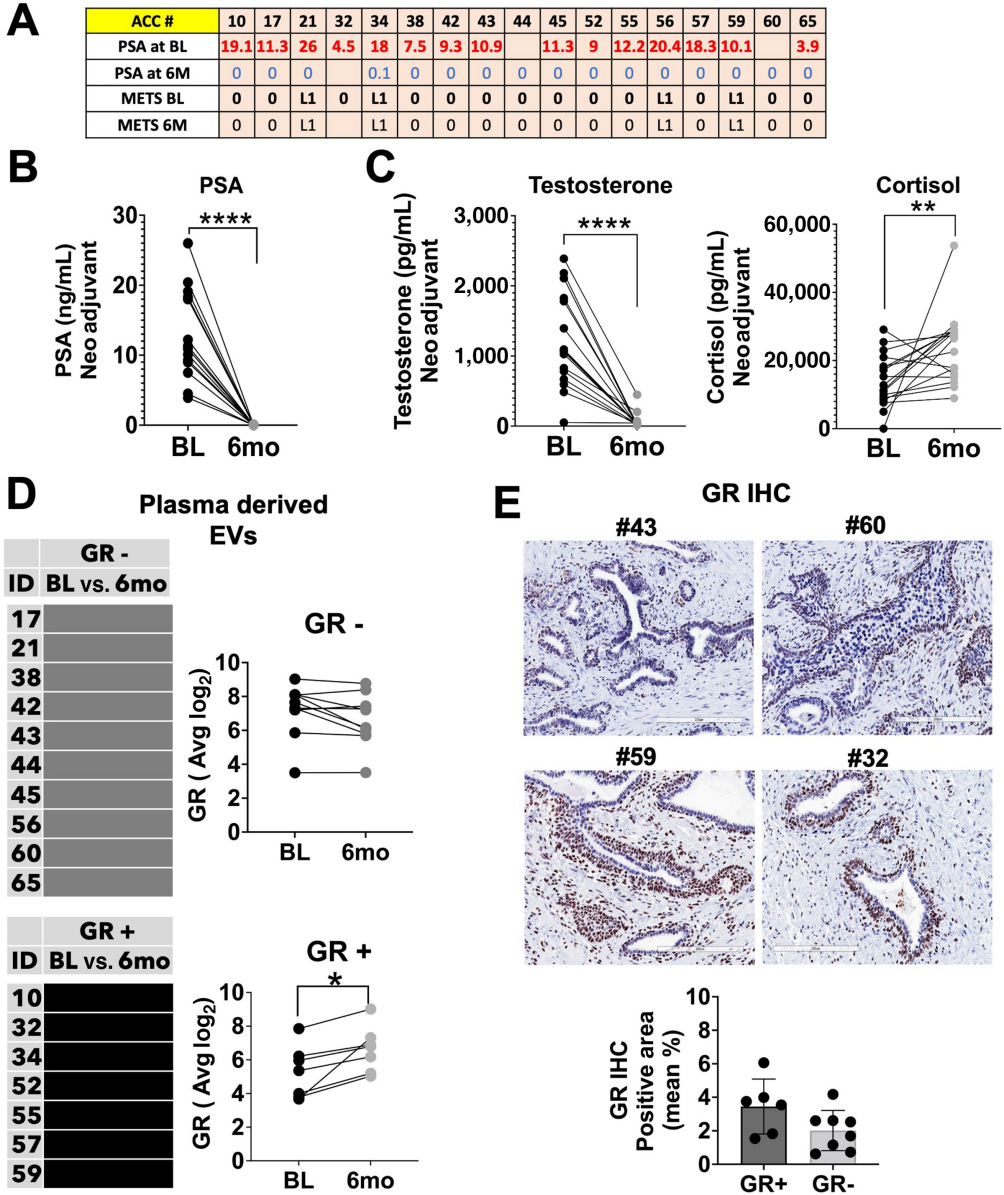
Monitoring changes in GR signaling in patients with localized hormone naïve disease treated with ADT. **A,** Preoperative trial NCT03279250 Clinical data and PSA concentration at BL and after 6 months of treatments (6 months). **B,** PSA and steroid levels in plasma, PSA (****, *P* <0.0001), testosterone (****, *P* <0.0001). **C,** Steroid levels in plasma cortisol (**, *P* = 0.0045). **D,** GR expression in RNA from EVs transcriptome analysis at BL and after 6 months of treatment. Patients were divided in two different groups bases on upregulation >0.5 or downregulation <0.5 of GR expression. Differential GR expression in GR− and GR+ groups from transcriptome analysis in plasma-derived EVs: GR+ (*, *P* = 0.0125). **E,** GR expression in tissues collected from patient's prostatectomy after therapy. IHC images from selected patients GR− and GR+ and IHC quantification of all GR+ and GR− patients with ImageJ.

To examine the feasibility of EVs GR detection in the clinical setting, we used patient plasma samples collected before and after 6 months ADT and ASI. In this clinical trial (NCT03279250), patients with localized high-risk or unfavorable intermediate-risk prostate cancer were treated with LHRH analog plus apalutamide (ARM A) for 6 months prior to radical prostatectomy. Plasma samples were collected before and after 6-month treatment. RNA was extracted from EVs, and total transcriptome profiling was performed by Clariom D for selected genes (Fig. 6). The PSA became undetectable or nearly undetectable in all men included in this analysis (Fig. 6A and B). GSEA showed changes in the Androgen Response pathway with a normalized enrichment score of −2.3, *P* value 0.007 (Supplementary Fig. S5A–S5C). Steroids levels analysis revealed a significant decrease in testosterone concentration and an increase in cortisol levels (Fig. 6C). We identified two subgroups of patients, those whose GR levels in EVs increased in response to ADT + ASI (GR+, 7 patients) and those that did not (GR−, 10 patients) as determined by the Clariom D Transcriptome Array (Fig. 6D). The GR-responsive genes PGK1, ALOX5AP, and CHCHD7 showed significant increase in expression only in the GR+ group (Supplementary Fig. S5D). To correlate the observed changes in GR expression in EVs with tumor tissue, prostatectomy samples from the same patients were stained for GR protein (Fig. 6E). Representative IHC images of 4 selected patients from the GR+ group (patient 59 and 32) and the GR− group (patient 43 and 60) demonstrated presence of GR expression in prostate cancer tumor cells, whereas lower GR expression was observed in the patients from the GR− group (Fig. 6E). Quantification of GR expression in tissues staining for all patients GR− and GR+, revealed a good correlation between expression of GR mRNA in EVs and expression of GR protein in tissue biopsies (Fig. 6E).

## Discussion

In this study, we explored the utility of analyzing GR expression in EVs from liquid biopsies to monitor the acquisition of resistance to ASI in prostate cancer. We showed that the dynamic changes to AR signaling inhibition can be detected in plasma-derived EVs as evidenced by changes in KLK3 (PSA) and GR mRNA in two different preclinical models, LNCaP cells and the MDA PCa 322-2-6a PDX. Importantly, in mice responding to ENZA treatment, GR expression was not induced at 14 days and remained low until the end of the experiment. In contrast, in the mice with ENZA-resistant tumor, GR induction was detected in the plasma-derived EVs early as 14 days posttreatment and continually increased until the end of the experiment. These data suggest that GR upregulation is an early event in the prostate cancer cells developing resistance to ASI.

Tissue biopsy is remaining the gold to diagnose cancer but using serial tissue biopsies to monitor response and elucidate mechanism during treatment poses significant challenges, including their relative invasiveness (16). In contrast, our study suggests that liquid biopsy analysis of plasma-derived EVs can longitudinally monitor changes in the tumor microenvironment to inform mechanism of therapy resistance to ASI and suggest novel therapy strategies. The potential of EVs analysis is particularly salient in advanced prostate cancer where bone metastases are predominant, because bone specimen frequently yield insufficient tumor content and the decalcification processing required to analyze bone specimens interferes with many molecular pathology assays, including IHC and DNA/RNA sequencing (17–19). Ongoing efforts to further refine EVs as diagnostic, prognostic, and predictive biomarkers include the development of techniques to isolate cell-specific EVs (16, 20).

Elevated GR in the tumors has been shown to correlate with poor prognosis (12, 21), and the ability to monitor GR levels in the plasma-derived EVs may have clinical implications. It has been previously shown that elevated GR in tumors may be due to GR playing a role in tumor progression (22, 23). GR regulates many genes that are important for different physiologic function such as catabolism, inflammation, and apoptosis/cell viability (24). In a subset of patients with prostate cancer, it has been shown that GR expression, which normally is low, increase after ASI and that GR induction is associated with tumor growth and disease progression (25). Patients whose bone metastases expressed relatively high GR after treatment with ENZA are less likely to achieve long-term benefit from the treatment (2, 12). These observations suggest that GR may play a role in tumor progression. The coclinical findings in our study indicate that GR is upregulated early in response to ASI as a mechanism of acquired resistance and that targeting GR in these patients leads to further tumor inhibition.

In this study, we used mifepristone (RU486), a GR inhibitor has gained great interest regarding its potential use as an anticancer agent where it has demonstrated significant growth inhibition and antitumor effects on various human cancer cell lines (26, 27). While the addition of mifepristone to ENZA inhibited tumor growth in ENZA-resistant tumors, the effect was transient. Our data suggest that GR-independent resistance mechanisms quickly evolve in response to GR inhibition. In concordance with our findings, a randomized phase I/II trial of ENZA and mifepristone combination for metastatic castration-resistant prostate cancer (PMID 35110415) was safe and well tolerated but did not meet its primary endpoint to delaying time to PSA, radiographic or clinical PFS and was terminated early (28). Clinical trials evaluating more selective inhibitors of GR are currently ongoing and will provide further insight into the clinical significance of GR as a resistance pathway to ASI (NCT03928314).

Prostate cancer is a heterogeneous disease, from a clinical, morphologic, and molecular perspective (29, 30). Numerous reports have documented that >80% of primary prostate cancers show multiple distinct tumor foci (31, 32). Sequencing efforts have demonstrated a high level of genomic diversity between different patients and within individual patients at the level of the distinct foci within the primary tumor between different metastatic sites (30, 33, 34). The clinical challenges posed by the complex molecular heterogeneity of prostate cancer requires developing tools to deconvolute biology to arrive at personalized treatment strategies. Transcriptome analysis of RNA from EVs, showed heterogeneity in response to ASI in the preclinical models and in the clinical trials analyzed. In the preclinical model, we show differential response to ASI in both LNCaP and MDA PCa 322-2-6a models with some tumors responding to ASI and some not. It has been previously shown that LNCaP cells have heterogeneity in their responses to ADT because of the microsatellite instability and mismatch repair defects (30, 31). By using human-GR sequence-specific primers, we could monitor specific changes associated with the human prostate cancer cells and not the host (mouse). It would be of interest to investigate the changes of the host GR in response to ENZA and whether it is also a cause of resistance to this therapy.

In the preoperative trial, the patient samples also showed heterogeneity in their GR expression in EVs that led to a substratification of the patients in two different groups according to their changes in EV GR levels following ADT treatment. Transcriptome analysis of RNA from EVs isolated from individual patients, demonstrated that GR induction occurs in a subset of patients. The observed GR induction was associated with expression of GR-responsive gene such as PGK1, ALOX5AP, and CHCHD7 indicating activation of the GR signaling pathway (35, 36) The possibility to monitor GR signaling in liquid biopsies though EVs can be instrumental because it will inform development of resistance to ADT and potentially inform for a timely intervention with a GR inhibitor or other therapies. In addition, the transcriptome analysis provided additional molecular information that may improve our understanding on the mechanisms of GR induction, as well as potential additional mechanisms of resistance to ADT. It follows that assessing GR levels serially following ENZA treatment by performing qRT-PCR in plasma-derived EVs may identify the patients that have acquired resistance to ASI and inform of potential treatment strategies (37, 38).

In conclusion, AR and GR expression can be longitudinally monitored with plasma-derived EVs during ENZA treatment *in vitro*, *in vivo*, and in patient samples. These findings validate that GR expression in EVs detects dynamic changes that reflect changes in the tumor cell, correlate with tumor growth and acquisition of resistance to ENZA and may inform rational precision therapy.

## Supplementary Material

Supplementary Figure 1 Cells and EVs characterization.

Supplementary Figure 2 Cellular and EV RNA integrity

Supplementary Figure 3 GR knockdown

Supplementary Figure 4 GR inhibition in vivo

Supplementary Figure 5 Patient-derived EV transcriptome

## References

[1] Lianidou E , Pantel K. Liquid biopsies. Genes Chromosomes Cancer 2019;58:219–32. 30382599 10.1002/gcc.22695

[2] Arora VK , Schenkein E, Murali R, Subudhi SK, Wongvipat J, Balbas MD, . Glucocorticoid receptor confers resistance to antiandrogens by bypassing androgen receptor blockade. Cell 2013;155:1309–22. 24315100 10.1016/j.cell.2013.11.012 PMC3932525

[3] Gao Z , Pang B, Li J, Gao N, Fan T, Li Y. Emerging role of exosomes in liquid biopsy for monitoring prostate cancer invasion and metastasis. Front Cell Dev Biol 2021;9:679527. 34017837 10.3389/fcell.2021.679527 PMC8129505

[4] Kharaziha P , Ceder S, Li Q, Panaretakis T. Tumor cell-derived exosomes: a message in a bottle. Biochim Biophys Acta 2012;1826:103–11. 22503823 10.1016/j.bbcan.2012.03.006

[5] Vardaki I , Sanchez C, Fonseca P, Olsson M, Chioureas D, Rassidakis G, . Caspase-3-dependent cleavage of Bcl-xL in the stroma exosomes is required for their uptake by hematological malignant cells. Blood 2016;128:2655–65. 27742710 10.1182/blood-2016-05-715961

[6] Gulei D , Petrut B, Tigu AB, Onaciu A, Fischer-Fodor E, Atanasov AG, . Exosomes at a glance - common nominators for cancer hallmarks and novel diagnosis tools. Crit Rev Biochem Mol Biol 2018;53:564–77. 30247075 10.1080/10409238.2018.1508276

[7] Lianidou ES , Strati A, Markou A. Circulating tumor cells as promising novel biomarkers in solid cancers. Crit Rev Clin Lab Sci 2014;51:160–71. 24641350 10.3109/10408363.2014.896316

[8] Colombo M , Raposo G, Thery C. Biogenesis, secretion, and intercellular interactions of exosomes and other extracellular vesicles. Annu Rev Cell Dev Biol 2014;30:255–89. 25288114 10.1146/annurev-cellbio-101512-122326

[9] Vardaki I , Corn P, Gentile E, Song JH, Madan N, Hoang A, . Radium-223 treatment increases immune checkpoint expression in extracellular vesicles from the metastatic prostate cancer bone microenvironment. Clin Cancer Res 2021;27:3253–64. 33753455 10.1158/1078-0432.CCR-20-4790 PMC8172463

[10] Del Re M , Biasco E, Crucitta S, Derosa L, Rofi E, Orlandini C, . The detection of androgen receptor splice variant 7 in plasma-derived exosomal RNA strongly predicts resistance to hormonal therapy in metastatic prostate cancer patients. Eur Urol 2017;71:680–7. 27733296 10.1016/j.eururo.2016.08.012

[11] Decker KF , Zheng D, He Y, Bowman T, Edwards JR, Jia L. Persistent androgen receptor-mediated transcription in castration-resistant prostate cancer under androgen-deprived conditions. Nucleic Acids Res 2012;40:10765–79. 23019221 10.1093/nar/gks888 PMC3510497

[12] Puhr M , Hoefer J, Eigentler A, Ploner C, Handle F, Schaefer G, . The glucocorticoid receptor is a key player for prostate cancer cell survival and a target for improved antiandrogen therapy. Clin Cancer Res 2018;24:927–38. 29158269 10.1158/1078-0432.CCR-17-0989

[13] Yang Y , Li X, Mamouni K, Kucuk O, Wu D. Mifepristone has limited activity to enhance the *in vivo* efficacy of docetaxel and enzalutamide against bone metastatic and castration-resistant prostate cancer. Anticancer Res 2017;37:6235–43. 29061806 10.21873/anticanres.12074

[14] Zhang L , Hapon MB, Goyeneche AA, Srinivasan R, Gamarra-Luques CD, Callegari EA, . Mifepristone increases mRNA translation rate, triggers the unfolded protein response, increases autophagic flux, and kills ovarian cancer cells in combination with proteasome or lysosome inhibitors. Mol Oncol 2016;10:1099–117. 27233943 10.1016/j.molonc.2016.05.001 PMC5240778

[15] Kapperman HE , Goyeneche AA, Telleria CM. Mifepristone inhibits non-small cell lung carcinoma cellular escape from DNA damaging cisplatin. Cancer Cell Int 2018;18:185. 30479564 10.1186/s12935-018-0683-z PMC6238342

[16] Yu W , Hurley J, Roberts D, Chakrabortty SK, Enderle D, Noerholm M, . Exosome-based liquid biopsies in cancer: opportunities and challenges. Ann Oncol 2021;32:466–77. 33548389 10.1016/j.annonc.2021.01.074 PMC8268076

[17] Li X , Corbett AL, Taatizadeh E, Tasnim N, Little JP, Garnis C, . Challenges and opportunities in exosome research-perspectives from biology, engineering, and cancer therapy. APL Bioeng 2019;3:011503. 31069333 10.1063/1.5087122 PMC6481742

[18] Li P , Kaslan M, Lee SH, Yao J, Gao Z. Progress in exosome isolation techniques. Theranostics 2017;7:789–804. 28255367 10.7150/thno.18133 PMC5327650

[19] Liu J , Ren L, Li S, Li W, Zheng X, Yang Y, . The biology, function, and applications of exosomes in cancer. Acta Pharm Sin B 2021;11:2783–97. 34589397 10.1016/j.apsb.2021.01.001 PMC8463268

[20] Willms E , Johansson HJ, Mager I, Lee Y, Blomberg KE, Sadik M, . Cells release subpopulations of exosomes with distinct molecular and biological properties. Sci Rep 2016;6:22519. 26931825 10.1038/srep22519 PMC4773763

[21] Pan D , Kocherginsky M, Conzen SD. Activation of the glucocorticoid receptor is associated with poor prognosis in estrogen receptor-negative breast cancer. Cancer Res 2011;71:6360–70. 21868756 10.1158/0008-5472.CAN-11-0362 PMC3514452

[22] Fakih M , Johnson CS, Trump DL. Glucocorticoids and treatment of prostate cancer: a preclinical and clinical review. Urology 2002;60:553–61. 12385906 10.1016/s0090-4295(02)01741-7

[23] Isikbay M , Otto K, Kregel S, Kach J, Cai Y, Vander Griend DJ, . Glucocorticoid receptor activity contributes to resistance to androgen-targeted therapy in prostate cancer. Horm Cancer 2014;5:72–89. 24615402 10.1007/s12672-014-0173-2 PMC4440041

[24] Timmermans S , Souffriau J, Libert C. A general introduction to glucocorticoid biology. Front Immunol 2019;10:1545. 31333672 10.3389/fimmu.2019.01545 PMC6621919

[25] Sahu B , Laakso M, Pihlajamaa P, Ovaska K, Sinielnikov I, Hautaniemi S, . FoxA1 specifies unique androgen and glucocorticoid receptor binding events in prostate cancer cells. Cancer Res 2013;73:1570–80. 23269278 10.1158/0008-5472.CAN-12-2350

[26] Chen J , Wang J, Shao J, Gao Y, Xu J, Yu S, . The unique pharmacological characteristics of mifepristone (RU486): from terminating pregnancy to preventing cancer metastasis. Med Res Rev 2014;34:979–1000. 24585714 10.1002/med.21311

[27] Taplin ME , Manola J, Oh WK, Kantoff PW, Bubley GJ, Smith M, . A phase II study of mifepristone (RU-486) in castration-resistant prostate cancer, with a correlative assessment of androgen-related hormones. BJU Int 2008;101:1084–9. 18399827 10.1111/j.1464-410X.2008.07509.x

[28] Serritella AV , Shevrin D, Heath EI, Wade JL, Martinez E, Anderson A, . Phase I/II trial of enzalutamide and mifepristone, a glucocorticoid receptor antagonist, for metastatic castration-resistant prostate cancer. Clin Cancer Res 2022;28:1549–59. 35110415 10.1158/1078-0432.CCR-21-4049 PMC9012680

[29] Haffner MC , Zwart W, Roudier MP, True LD, Nelson WG, Epstein JI, . Genomic and phenotypic heterogeneity in prostate cancer. Nat Rev Urol 2021;18:79–92. 33328650 10.1038/s41585-020-00400-w PMC7969494

[30] Tang DG . Understanding and targeting prostate cancer cell heterogeneity and plasticity. Semin Cancer Biol 2022;82:68–93. 34844845 10.1016/j.semcancer.2021.11.001 PMC9106849

[31] Alizadeh AA , Aranda V, Bardelli A, Blanpain C, Bock C, Borowski C, . Toward understanding and exploiting tumor heterogeneity. Nat Med 2015;21:846–53. 26248267 10.1038/nm.3915 PMC4785013

[32] Gerlinger M , Catto JW, Orntoft TF, Real FX, Zwarthoff EC, Swanton C. Intratumour heterogeneity in urologic cancers: from molecular evidence to clinical implications. Eur Urol 2015;67:729–37. 24836153 10.1016/j.eururo.2014.04.014

[33] Spratt DE , Zumsteg ZS, Feng FY, Tomlins SA. Translational and clinical implications of the genetic landscape of prostate cancer. Nat Rev Clin Oncol 2016;13:597–610. 27245282 10.1038/nrclinonc.2016.76 PMC5030163

[34] Andreoiu M , Cheng L. Multifocal prostate cancer: biologic, prognostic, and therapeutic implications. Hum Pathol 2010;41:781–93. 20466122 10.1016/j.humpath.2010.02.011

[35] Jung Y , Shiozawa Y, Wang J, Wang J, Wang Z, Pedersen EA, . Expression of PGK1 by prostate cancer cells induces bone formation. Mol Cancer Res 2009;7:1595–604. 19825988 10.1158/1541-7786.MCR-09-0072 PMC2771860

[36] Puhr M , Eigentler A, Handle F, Hackl H, Ploner C, Heidegger I, . Targeting the glucocorticoid receptor signature gene Mono Amine Oxidase-A enhances the efficacy of chemo- and anti-androgen therapy in advanced prostate cancer. Oncogene 2021;40:3087–100. 33795839 10.1038/s41388-021-01754-0 PMC8084733

[37] Kumar R . Emerging role of glucocorticoid receptor in castration resistant prostate cancer: a potential therapeutic target. J Cancer 2020;11:696–701. 31942193 10.7150/jca.32497 PMC6959034

[38] Bakour N , Moriarty F, Moore G, Robson T, Annett SL. Prognostic significance of glucocorticoid receptor expression in cancer: a systematic review and meta-analysis. Cancers 2021;13:1649. 33916028 10.3390/cancers13071649 PMC8037088

