# Supplementary material for: Monitoring Glucocorticoid Receptor in Plasma-derived Extracellular Vesicles as a Marker of Resistance to Androgen Receptor Signaling Inhibition in Prostate Cancer
Source: Cancer Res Commun. 2023 Dec 13;3(12):2531–43. doi: 10.1158/2767-9764.CRC-23-0362 (PMC10718063; doi:10.1158/2767-9764.CRC-23-0362)
Supplement: Supplementary Figure 1 — Cells and EVs characterization. [file crc-23-0362-s01.pdf]

Supplementary Figure 1

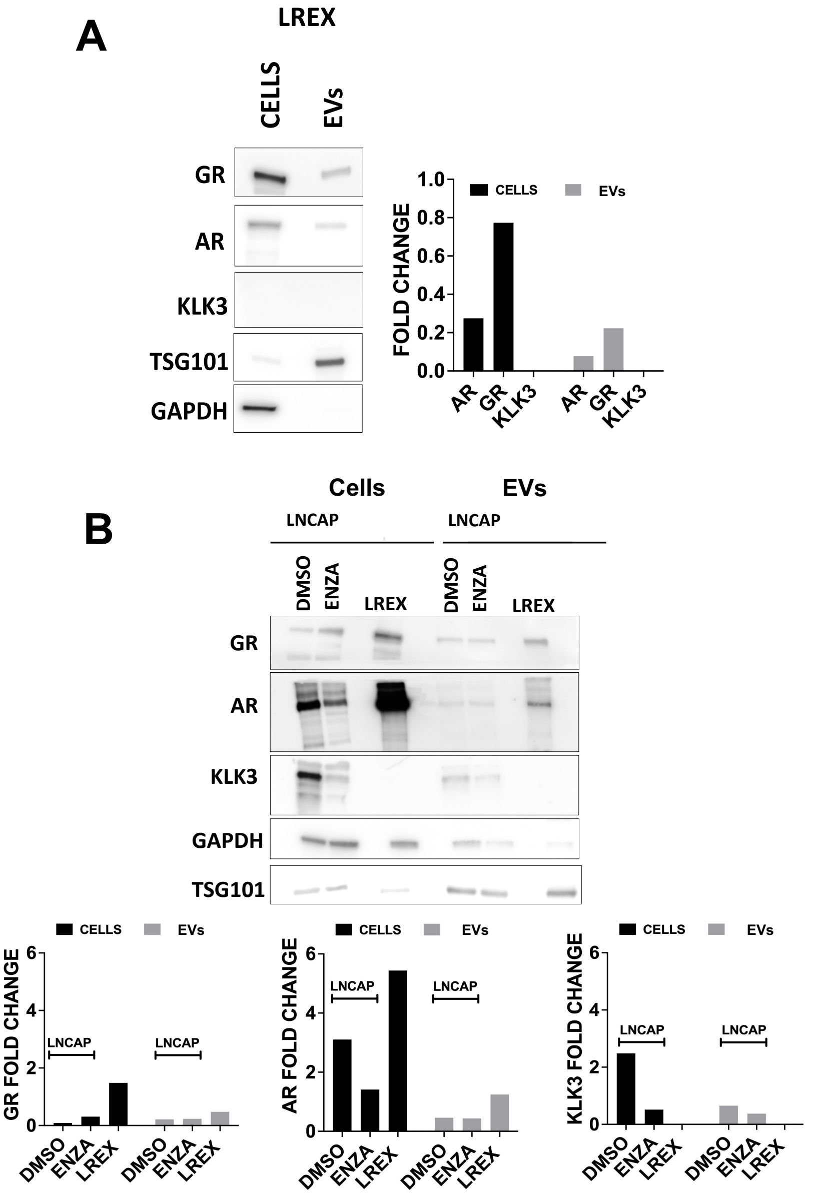

Cells and EVs characterization. (A) Immunoblot analysis for GR, AR and KLK3 in LREX cells and EVs (TSG101 EVs loading control and GAPDH cells loading control). **(B)** Immunoblot analysis for GR, AR and KLK3 in LNCaP cells and EVs after treatment for 5 days with 1μM ENZA. TSG101 EVs loading control and GAPDH cells loading control. LREX cells were used as control for GR expression.
