# Supplementary material for: Monitoring Glucocorticoid Receptor in Plasma-derived Extracellular Vesicles as a Marker of Resistance to Androgen Receptor Signaling Inhibition in Prostate Cancer
Source: Cancer Res Commun. 2023 Dec 13;3(12):2531–43. doi: 10.1158/2767-9764.CRC-23-0362 (PMC10718063; doi:10.1158/2767-9764.CRC-23-0362)
Supplement: Supplementary Figure 2 — Cellular and EV RNA integrity [file crc-23-0362-s02.pdf]

Supplementary Figure 2

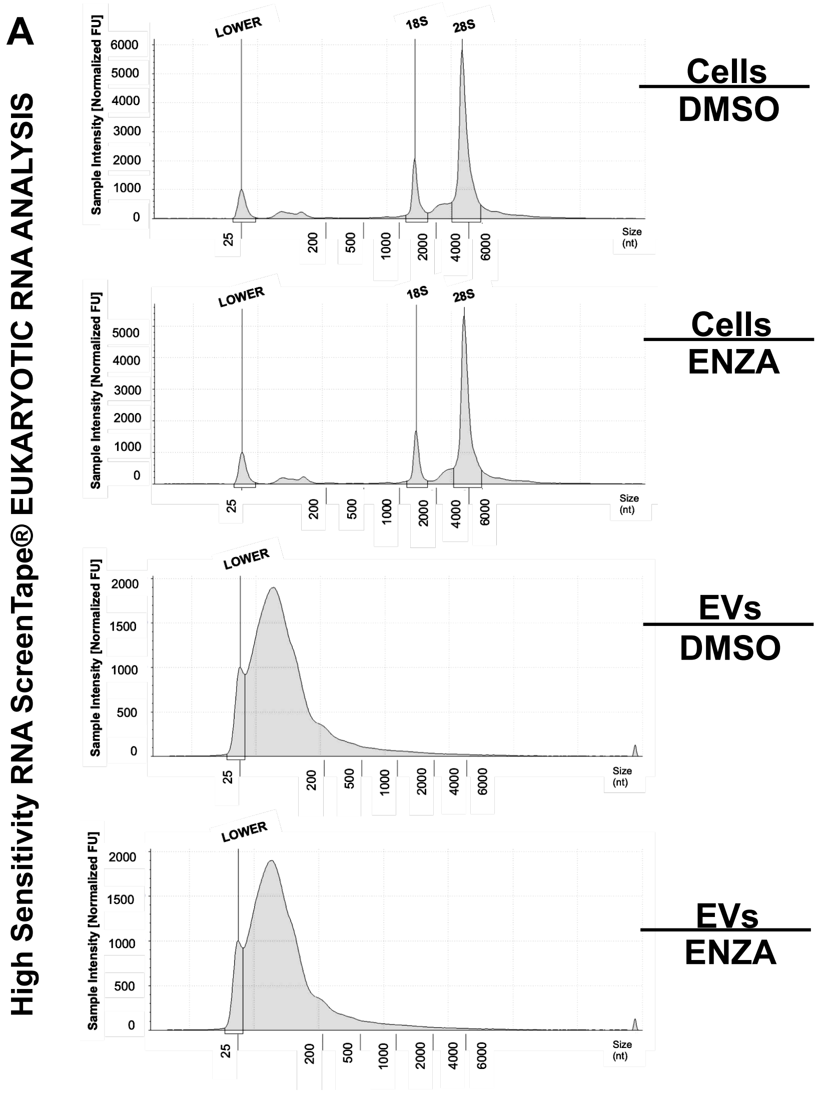

High Sensitivity RNA ScreenTape® EUKARYOTIC RNA ANALYSIS. (A) Separation of total RNA samples in eukaryotic samples. Difference in quality, quantity, and sizing between treated (ENZA) and untreated (DMSO) cells and treated (ENZA) and untreated (DMSO) cell derived EVs.
