# Supplementary material for: Monitoring Glucocorticoid Receptor in Plasma-derived Extracellular Vesicles as a Marker of Resistance to Androgen Receptor Signaling Inhibition in Prostate Cancer
Source: Cancer Res Commun. 2023 Dec 13;3(12):2531–43. doi: 10.1158/2767-9764.CRC-23-0362 (PMC10718063; doi:10.1158/2767-9764.CRC-23-0362)
Supplement: Supplementary Figure 3 — GR knockdown [file crc-23-0362-s03.pdf]

Supplementary Figure 3

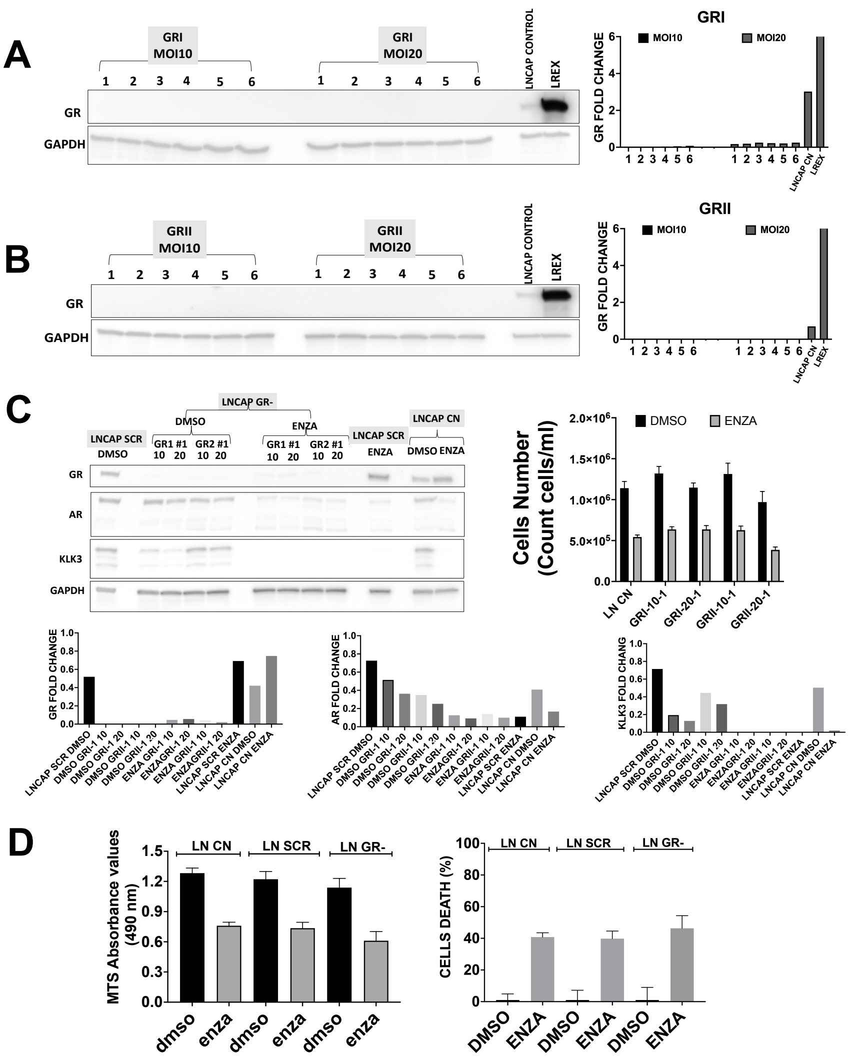

GR knockdown *in vitro.* (A) Immunoblot analysis of the indicated proteins in LNCaP cells after transduction with shGR-I (GRI) and **(B)** shGR-II (GRII) at different MOI (LREX were used as GR expression control and parental LNCaP cells as control). **(C)** Immunoblot analysis of the indicated proteins and cells count in LNCaP cells transduced with GRI and GRII at different MOI and treated for 5 days with 1µM ENZA and DMSO as vehicle control (LNCaP SCR and parental LNCaP cells were treated as well and used as control). **(D)** Cells viability and cells death evaluation on LNCaP cell GR knockdown (GRII-) after treatment for 5 days with 1µM ENZA.
