# Supplementary material for: Monitoring Glucocorticoid Receptor in Plasma-derived Extracellular Vesicles as a Marker of Resistance to Androgen Receptor Signaling Inhibition in Prostate Cancer
Source: Cancer Res Commun. 2023 Dec 13;3(12):2531–43. doi: 10.1158/2767-9764.CRC-23-0362 (PMC10718063; doi:10.1158/2767-9764.CRC-23-0362)
Supplement: Supplementary Figure 4 — GR inhibition in vivo [file crc-23-0362-s04.pdf]

**Supplementary Figure 4**

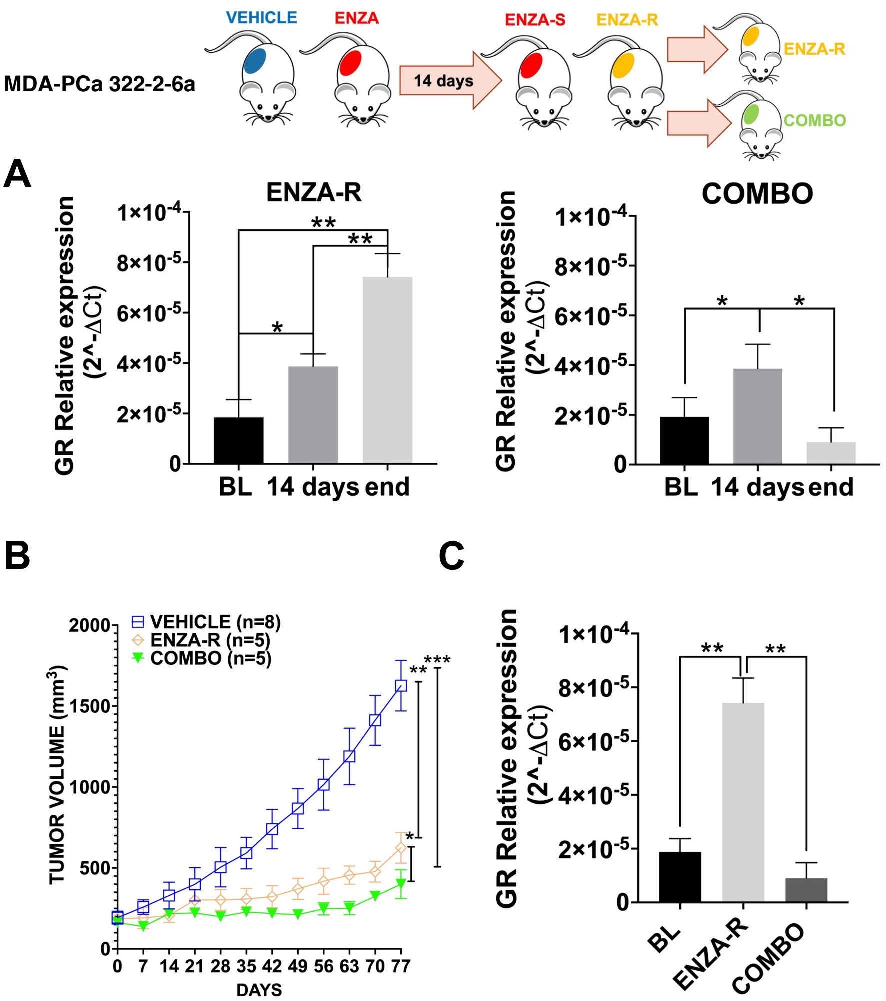


**Effect of GR inhibitor *in vivo* on MDA PCa 322-2-6a.** Schema and treatment planning for *in vivo* experiment on MDA PCa 322-2-6a. **(A)** Longitudinal analysis of ENZA-R group (14 days vs BL *0.0114 p-value; end vs BL **0.0063 p-value; end vs 14 days **0.0067 p-value) and COMBO group (14 days vs BL *0.0374 p-value; end vs 14 days *0.0322 p-value) BL, 14 days, and END. **(B)** Final tumor growth analysis of VEHICLE, ENZA-R and COMBO at 77 days (ENZA-R vs COMBO *0.0157 p-value; COMBO vs VEHICLE** 0.0005 p-value; ENZA-R vs vehicle ** 0.0045 p-value). **(C)** RT-qPCR results of GR for ENZA-R and COMBO in plasma derived EVs at the end of the experiment comparing to BL (ENZA-R vs BL **0.0063 p-value; COMBO vs ENZA-R ** 0.0092 p-value).
