# Supplementary material for: Monitoring Glucocorticoid Receptor in Plasma-derived Extracellular Vesicles as a Marker of Resistance to Androgen Receptor Signaling Inhibition in Prostate Cancer
Source: Cancer Res Commun. 2023 Dec 13;3(12):2531–43. doi: 10.1158/2767-9764.CRC-23-0362 (PMC10718063; doi:10.1158/2767-9764.CRC-23-0362)
Supplement: Supplementary Figure 5 — Patient-derived EV transcriptome [file crc-23-0362-s05.pdf]

A **Top downregulated pathways (Hallmarks)**

GSEA Hallmarks Pathways	NES	p-val
HYPOXIA	-2.88797	0.000000
MITOTIC_SPINDLE	-2.73585	0.000333
EPITHELIAL_MESENCHYMAL_TRANSITION	-2.69983	0.000267
E2F_TARGETS	-2.69169	0.000222
MTORC1_SIGNALING	-2.53221	0.000591
G2M_CHECKPOINT	-2.41595	0.001287
**ANDROGEN_RESPONSE**	**-2.38543**	**0.001144**
PROTEIN_SECRETION	-2.36609	0.001180
TGF_BETA_SIGNALING	-2.23897	0.002663
ESTROGEN_RESPONSE_EARLY	-2.23422	0.002441
PI3K_AKT_MTOR_SIGNALING	-2.16224	0.003708
MYOGENESIS	-2.14518	0.003980
KRAS_SIGNALING_UP	-2.03548	0.006945
TNFA_SIGNALING_VIA_NFKB	-1.93260	0.013023

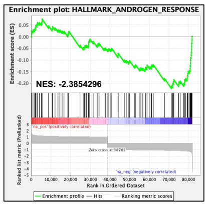

B **Top downregulated pathways (KEGG)**

GSEA KEGG Pathways	NES	p-val
ADHERENS_JUNCTION	-3.00023	0.00000
UBIQUITIN_MEDIATED_PROTEOLYSIS	-2.96807	0.00071
PATHWAYS_IN_CANCER	-2.57910	0.00271
OLFACTORY_TRANSDUCTION	-2.55755	0.00214
P53_SIGNALING_PATHWAY	-2.44790	0.00266
FOCAL_ADHESION	-2.41816	0.00301
TIGHT_JUNCTION	-2.28880	0.00532
REGULATION_OF_ACTIN_CYTOSKELETON	-2.26736	0.00595
APOPTOSIS	-2.26627	0.00558
PHOSPHATIDYLINOSITOL_SIGNALING_SYSTEM	-2.22366	0.00732
CELL_CYCLE	-2.19603	0.00842
ECM_RECEPTOR_INTERACTION	-2.17171	0.00964
**PROSTATE_CANCER**	**-2.16590**	**0.00948**
ENDOCYTOSIS	-2.14051	0.01056

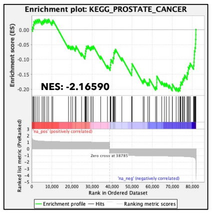

C

Ingenuity Canonical Pathways	z-score	-log(p-value)
Sirtuin Signaling Pathway	3.019	4.56
RhoA Signaling	3.015	1.50
Signaling by Rho Family GTPases	2.769	2.35
HER-2 Signaling in Breast Cancer	2.712	5.57
Remodeling of Epithelial Adherens Junctions	2.138	4.01
HGF Signaling	2.064	4.65
Cdc42 Signaling	2	2.41
Sumoylation Pathway	1.976	2.09
Pyridoxal 5'-phosphate Salvage Pathway	1.826	3.35
Inhibition of ARE-Mediated mRNA Degradation Pathway	1.786	3.34
fMLP Signaling in Neutrophils	1.769	4.84
Rac Signaling	1.677	2.70
CXCR4 Signaling	1.604	3.92
Neuregulin Signaling	1.567	1.93
ATM Signaling	1.543	4.94
Senescence Pathway	1.464	4.05
Aldosterone Signaling in Epithelial Cells	1.414	1.72
Role of CHK Proteins in Cell Cycle Checkpoint Control	1.4	3.82
**Glucocorticoid Receptor Signaling**	**1.354**	**2.37**
ErbB4 Signaling	1.347	2.83

D

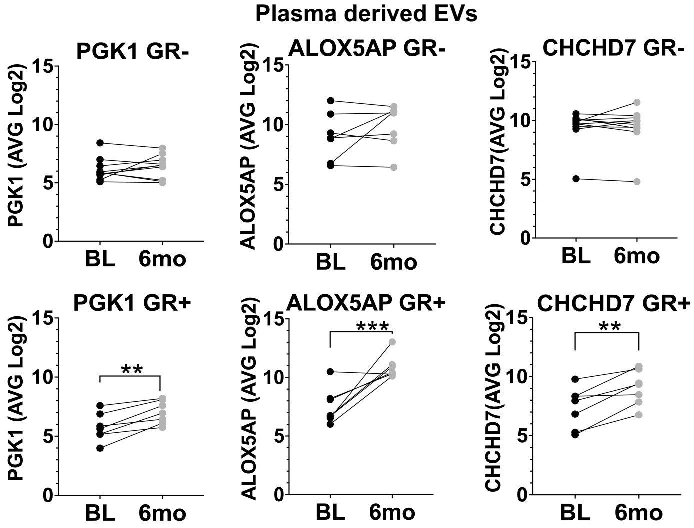

Clinical trials RNA from EVs results. (A) Transcriptome analysis for Neo-adjuvant patient' s RNA from EVs, top downregulated pathway (HALLMARKS). **(B)**Transcriptome analysis for Neo-adjuvant patient' s RNA from EVs, top downregulated pathway (KEGG). **(C)** Table of Ingenuity canonical pathway. **(D)** GR responsive genes from Transcriptome analysis in plasma derived EVs, the patients were divided in GR- and GR+, significant genes: PGK1 (**0.0028 p-value); ALOX5AP (***p-value: 0.0004); CHCHD7 (**p-value: 0.0043).
